# A New Record Diagram

**Published:** 1887-06

**Authors:** 


### A New Record Diagram.

We have just recieved a diagram, prepared by Dr. Louis Ottofy, of Chicago, the object of which is to enable the dentist to make a complete record, both historical and diagnostic of the mouth and teeth of those who come under his care.

Appropriate columns are given for recording the parentage, the nativity, the constitutional peculiarities of the patient; also the condition of the saliva in its various states, and the various phases of disease that may be found to exist in the mouth or teeth. This diagram is the most complete thing of the kind we have ever seen; and should be in the hands of every dentist who has any interest in keeping such records; and this every dentist ought to do; and study them, too, not only himself but make them available to his professional brethren as well.

There are important lessons to be learned in almost every case that is presented, if we will but learn them; and in order to make them serve any valuable purpose, they must be made a matter of record, and the records studied. Had such record and study been followed closely by any considerable portion of the profession for the last twenty-five years, decay of the teeth, salivary calculus and pyorrhoea alveolarus, etc., etc., would not to-day, be so obscure to the mass of the profession as they unfortunately are.

We can most heartily suggest to all the readers of the REGISTER to send to Dr. Ottofy for a sample sheet of his register; and there is hardly a doubt but that it will recieve the approval of every one.

Every step that looks to the improvement and elevation of our profession, should receive the hearty approbation of every dentist throughout the country.

